# Brooke-Spiegler Syndrome

**Published:** 2018-07-23

**Authors:** Waseem Mohiuddin, Jake Laun, Wayne Cruse

**Affiliations:** ^a^Division of Plastic Surgery, Department of Surgery, Thomas Jefferson University Hospital, Philadelphia, Pa; ^b^Department of Plastic Surgery, Morsani College of Medicine, Tampa, Fla

**Keywords:** Brooke-Spiegler syndrome, trichoepithelioma, spiradenoma, cylindroma, adnexal tumors

**Figure:**
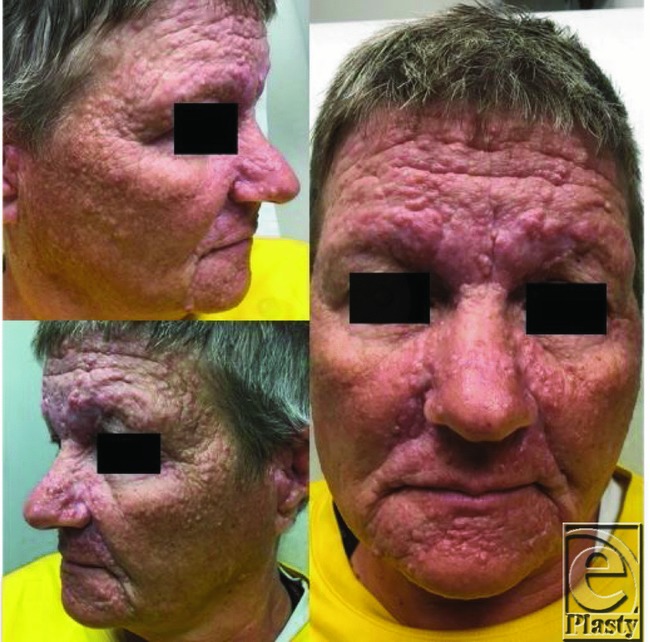


## DESCRIPTION

A 52-year-old woman presented to the cutaneous clinic with multiple firm, rubbery, flesh-colored papules localized to the face. Biopsy confirmed trichoepitheliomas. Because of clinical and histological manifestations, the patient was suspected to have Brooke-Spiegler syndrome. Patient stated that she had had these lesions since she was a teenager and had had them biopsied in the past, with some shown to be basal cell carcinomas.

## QUESTIONS

1. What is Brooke-Spiegler syndrome?
2. How is Brooke-Spiegler syndrome diagnosed?
3. What are the treatment options for Brooke-Spiegler syndrome?
4. What is the prognosis for Brooke-Spiegler syndrome?

## DISCUSSION

Brooke-Spiegler syndrome is a rare autosomal dominant condition associated with numerous nodular, adnexal tumors localized to the face, scalp, and neck, with less frequent involvement of the trunk and extremities.^1^^-^4 Described in 1892 and 1899 by Brooke and Spiegler, patients typically present in late childhood and early adolescence.^3^^,^^5^ It predominantly affects women, with the male to female ratio of 1:6-9.6, and exact incidence is unknown.^6^ Most nodules are 0.5 to 3 cm in size, although larger lesions may occasionally be seen.^4^ Histologically, these nodules correspond to spiradenomas, cylindromas, and trichoepitheliomas. Multiple familial trichoepitheliomas are a phenotypic variant of the disease that is characterized by numerous trichoepitheliomas without the presence of other adnexal tumors. These trichoepitheliomas tend to be localized to the nasolabial fold and inner aspects of the eyebrows.^4^ A second variant, familial cylindromatosis, is characterized by the presence of numerous cylindromas.^7^ Scalp cylindromas may become numerous enough to eventually cover the entire scalp and result in hair loss (referred to as “turban tumor”).^3^

In those suspected of the disease, biopsy specimens are warranted to aid in the diagnosis of Brooke-Spiegler syndrome. While most lesions are histologically pure, neoplasms with hybrid features, such as spiradenocylindromas, spiradenoma-trichoepitheliomas, cylindroma-trichoepitheliomas, or the concurrence of all 3 tumors in 1 lesion, may also be seen.^3^ The *CYLD* tumor suppressor gene on chromosome 16 has been implicated in the pathogenesis of Brooke-Spiegler syndrome and its phenotypic variants.^1^^,^^5^ Genetic testing for a mutation in this gene may confirm the diagnosis. Biopsies are important, as there can also be transformation to malignancy including basal cell carcinomas.^6^

Given the rarity of Brooke-Spiegler syndrome, treatment is controversial. Successful treatment has been described with dermabrasion, electrosurgery, cryosurgery, radiosurgery, ablation with neodymium-doped YAG, erbium:YAG, or carbon dioxide lasers, and photodynamic therapy.^2^^,^^3^^,^^7^ Medical treatment options include sodium salicylate and prostaglandin A1, a combination of aspirin and adalimumab, and topical imiquimod.^2^^,^^3^ There have also been some discussions of utilization of systemic chemotherapy or sonic hedgehog inhibitors such as vismodegib, which our patient used with some initial success. While surgical excision is the mainstay of treatment of solitary tumors, the multifocal nature of Brooke-Spiegler syndrome usually precludes this option, although the use of micrographic surgery and subgaleal scalp excision with split-thickness skin grafting has been reported with some success.^5^^,^^7^

Usually associated with rapid enlargement, ulceration, and bleeding, malignant transformation of lesions occurs in 5% to 10% of patients with Brooke-Spiegler syndrome.^4^ Close clinical follow-up is warranted to identify these changes. Extensive involvement of the eyelids and external auditory meatus may lead to blindness and deafness, respectively.^4^ In addition, patients have an increased risk of adenocarcinoma of the major and minor salivary glands, with parotid gland tumors most commonly reported.^1^^,^^4^

Brooke-Spiegler syndrome is a devastating condition with no widely accepted standard of treatment. Because Brooke-Spiegler syndrome is characterized by diffuse involvement and numerous adnexal tumors of the head and neck, surgical excision is often difficult. Common treatment options include electrosurgery, dermabrasion, and laser therapy. Close follow-up is essential to monitor for malignant transformation.
